# Histone deacetylase inhibitors enhance expression of NKG2D ligands in Ewing sarcoma and sensitize for natural killer cell-mediated cytolysis

**DOI:** 10.1186/2045-3329-2-8

**Published:** 2012-02-08

**Authors:** Dagmar Berghuis, Marco W Schilham, Hanneke I Vos, Susy J Santos, Stephan Kloess, Emilie P Buddingh', R Maarten Egeler, Pancras CW Hogendoorn, Arjan C Lankester

**Affiliations:** 1 Department of Pathology, Leiden University Medical Center, Leiden, The Netherlands; 2 Department of Pediatrics, Leiden University Medical Center, Leiden, The Netherlands; 3 Laboratory for Stem Cell Transplantation and Immunotherapy, Hospital of Johann Wolfgang Goethe-University, Frankfurt am Main, Germany

**Keywords:** Ewing sarcoma, natural killer cells, histone deacetylase inhibitor, combination immunotherapy, chemotherapy-resistance, tumour immunology

## Abstract

**Background:**

Ewing sarcoma patients have a poor prognosis despite multimodal therapy. Integration of combination immunotherapeutic strategies into first-/second-line regimens represents promising treatment options, particularly for patients with intrinsic or acquired resistance to conventional therapies. We evaluated the susceptibility of Ewing sarcoma to natural killer cell-based combination immunotherapy, by assessing the capacity of histone deacetylase inhibitors to improve immune recognition and sensitize for natural killer cell cytotoxicity.

**Methods:**

Using flow cytometry, ELISA and immunohistochemistry, expression of natural killer cell receptor ligands was assessed in chemotherapy-sensitive/-resistant Ewing sarcoma cell lines, plasma and tumours. Natural killer cell cytotoxicity was evaluated in Chromium release assays. Using ATM/ATR inhibitor caffeine, the contribution of the DNA damage response pathway to histone deacetylase inhibitor-induced ligand expression was assessed.

**Results:**

Despite comparable expression of natural killer cell receptor ligands, chemotherapy-resistant Ewing sarcoma exhibited reduced susceptibility to resting natural killer cells. Interleukin-15-activation of natural killer cells overcame this reduced sensitivity. Histone deacetylase inhibitor-pretreatment induced NKG2D-ligand expression in an ATM/ATR-dependent manner and sensitized for NKG2D-dependent cytotoxicity (2/4 cell lines). NKG2D-ligands were expressed *in vivo*, regardless of chemotherapy-response and disease stage. Soluble NKG2D-ligand plasma concentrations did not differ between patients and controls.

**Conclusion:**

Our data provide a rationale for combination immunotherapy involving immune effector and target cell manipulation in first-/second-line treatment regimens for Ewing sarcoma.

## Introduction

Ewing sarcoma is an aggressive round cell sarcoma characterized by specific gene fusions most commonly involving *TET *gene family products, though rarely other activating transcription factors [1-3]. It usually affects bone or soft tissue in children and young adults. Despite multimodal therapy consisting of high-dose chemotherapy, surgery and radiotherapy, survival of patients with Ewing sarcoma has not improved significantly during the past decade. Patients with therapy-resistant or metastatic Ewing sarcoma have the most unfavorable prognosis, with a 5-year overall survival of less than 30% [4-6], which has recently been demonstrated to be independent of Ewing sarcoma-ETS fusion type [4,5].

Natural killer (NK) cells are the main cytotoxic effector cells of the innate immune system, contributing to host anti-microbial and anti-tumour immune responses. In contrast to T-lymphocytes, these cells lack antigen-specific receptors. Instead, recognition of target cells and subsequent triggering of effector functions is regulated by integration of signals delivered from inhibitory (e.g. killer cell immunoglobulin receptors, CD94/NKG2A) and activating (e.g. natural killer group 2D (NKG2D), DNAX accessory molecule 1 (DNAM1), natural cytotoxicity receptor (NCR)) cell surface receptors [7,8]. Natural killer cells respond to several cytokines, including several interleukins and type I interferons, resulting in increased cytolytic, secretory and proliferative capacity [9]. Several studies have addressed the therapeutic potential and safety of immunotherapeutic/natural killer cell-based approaches for various cancer types, including sarcomas [10-15]. Sensitivity of tumours to natural killer cells critically depends on expression of ligands for activating receptors. Likewise, low expression of inhibitory human leukocyte antigen (HLA) class I molecules is an important prerequisite for successful natural killer cell triggering. Manipulation of either the balance between activating and inhibitory signals (by, for instance, e*x vivo *activation of immune cells) and/or sensitization of target cells for immune-mediated killing by combination immunotherapy may improve immunotherapeutic efficacy. For example, pre-conditioning of various cancer cell types by agents that activate the DNA damage response pathway may sensitize for natural killer cell cytotoxicity via induction of activating natural killer cell receptor ligands and/or death receptor expression. Comparable results have been observed for histone deacetylase inhibitors (HDI), which are currently emerging as potent anti-tumour agents [16-19].

Pre-clinical studies show that Ewing sarcoma can be targeted by (cytokine-activated) natural killer cells in a NKG2D-, DNAM1- and, as recently demonstrated, NCR-dependent manner [20-24]. Moreover, a potential role for natural killer cell alloreactivity in Ewing sarcoma disease control has recently been suggested [25]. Integration of natural killer cell-based (combination) immunotherapy into first-line treatment regimens or as a second-line approach represents a promising treatment option for Ewing sarcoma, in particular for patients with either intrinsic or acquired resistance to conventional therapies [26]. However, chemotherapy-resistance of Ewing sarcoma correlates with expression of genes involved in, among others, apoptosis signaling pathways [27]. Depending on the (apoptotic) pathways involved, resistance to apoptosis might render cells cross-resistant to immune cell cytotoxicity [28]. As yet, data about the susceptibility of therapy-resistant Ewing sarcoma to natural killer cell cytotoxicity are lacking. To obtain insight into the potential of natural killer cell-based combination immunotherapy for Ewing sarcoma, natural killer cell receptor ligand expression and susceptibility to natural killer cell cytotoxicity were evaluated in chemotherapy-sensitive and -resistant Ewing sarcoma tumour samples and cell lines respectively. Moreover, since HDI have the ability to exhibit direct cytotoxicity against Ewing sarcoma *in vitro *as well as *in vivo *and to sensitize for both conventional and more experimental treatment modalities [29-32], the potential of these agents to sensitize Ewing sarcoma for natural killer cell cytotoxicity was investigated.

## Methods

### Ewing sarcoma patients and samples

Peripheral blood samples from newly diagnosed Ewing sarcoma patients (2004-2009) were collected prior to start of chemotherapy (n = 27) and, in case of complete remission, six months after completion of therapy (n = 7) (treatment according to the EURO-EWING 99 trial [4]). Samples were centrifuged immediately and plasma was stored at -80°C until assayed. Mean age at diagnosis was 20.8 years (range 4-61 years). Plasma obtained from age-comparable healthy controls (n = 27; mean age 21.8, range 5-65 years) was used as a reference. Formalin-fixed paraffin-embedded, sequential pre-treatment, post-chemotherapy and recurrent metastatic tumour samples (n = 33) from eight Ewing sarcoma patients were obtained from the Department of Pathology, Leiden University Medical Center. In all cases, diagnosis was established according to WHO criteria including standard confirmatory immunohistochemistry and fusion transcript type. Follow-up provided information concerning initial tumour stage, (histological) chemotherapy response [33], recurrence rate and performance state. All patient material was coded, such that correlation with clinical data was only possible for physicians involved in treatment of the patients. Subsequent research was conducted following the ethical guidelines of the Dutch organization of scientific societies (FEDERA).

### Ewing sarcoma cell lines

Ewing sarcoma cell lines SK-ES-1, SK-N-MC, CADO-ES and STA-ET2.1 [34] were cultured in RPMI-1640 supplemented with streptomycin/penicillin (Invitrogen, Paisley, United Kingdom) and 10% fetal bovine serum ((FBS); Greiner Bio-One, Alphen a/d Rijn, The Netherlands). TC71 [34] and IOR/BER (kindly provided by dr. K. Scotlandi, Instituto Orthopedico Rizzoli, Bologna, Italy) were cultured in Iscove's Modified Dulbecco's Medium supplemented with streptomycin/penicillin and 10% FBS. Previously reported [27,35] 'natural' sensitivity of these cell lines to chemotherapeutic drugs currently used for treatment of Ewing sarcoma [4] was used to discriminate chemotherapy-sensitive (TC71, SK-ES-1, SK-N-MC) from chemotherapy-resistant (STA-ET2.1, CADO-ES, IOR/BER) cell lines. Molecular HLA typing of the cell lines, performed at Leiden University Medical Center (LUMC), was converted to serological equivalents (no serological equivalents exist for HLA-Cw*16 and -Cw*12): SK-ES-1 (A2/A11/B7/B44/Cw5/Cw7), SK-N-MC (A1/A25/B8/Cw7), CADO-ES (A11/A24/B15/B40/Cw4/Cw7), STA-ET-2.1 (A11/A24/B18/B40/Cw2/Cw5), TC71 (A2/A68/B15/B44/Cw3/Cw5), IOR/BER (A2/A11/B18/B51/Cw7/Cw15). The EBV B-LCL 107 cell line was generated from a healthy blood bank donor expressing HLA alleles which are ligands to all inhibitory killer cell immunoglobulin receptors [20]. The human erythroleukemia cell line K562 was obtained from American Type Culture Collection (Manassas, VA). Cell lines were routinely screened for mycoplasma contamination. Periodical authentication was performed by Short Tandem Repeat profiling and molecular HLA typing.

### Antibodies and reagents

The antibodies used for staining of antigens by flow cytometry and immunohistochemistry as well as for blocking of specific natural killer cell receptors and detection of soluble protein by ELISA are described in additional file 1, table S1. HDI [N-(2-amino-phenyl)-4-[N-(pyridine-3-ylmethoxycarbonyl)aminomethyl]benzamide] (MS-275) and suberoylanilide hydroxamic acid (SAHA) were purchased from Enzo Life Sciences (Raamsdonksveer, The Netherlands). Sodium butyrate (NaB) and the pharmacological ATM/ATR inhibitor caffeine were obtained from Sigma-Aldrich (Zwijndrecht, The Netherlands).

### Flow cytometric analysis of antigen surface expression

Flow cytometric analysis was performed on a FACScalibur (Beckton Dickinson, Franklin Lakes, NJ) and results were analyzed using Cellquest software, as previously described [36]. Ligand expression was represented as fold increase in Mean Fluorescence Intensity (MFI) over isotype control staining (MFI-ratio).

### Immunohistochemistry for detection of NKG2D ligand expression in Ewing sarcoma tumour samples

4-μm sections containing representative tumour were deparaffinized and citrate antigen retrieval was performed. Subsequent immunohistochemical stainings were performed and (semi-quantitatively) scored as previously described [36].

### Quantification of soluble MICA in Ewing sarcoma patient plasma samples

Concentrations of soluble MICA in plasma samples were determined by sandwich-ELISA using the MICA (human) detection set, according to the manufacturer's protocol (BAMOMAB/Axxora, Lörrach, Germany). In short, plates were coated overnight at 4°C with 5 μg/ml of the anti-MICA antibody (AMO1). After blocking with 15% BSA/PBS for 15 minutes at 37°C, plates were washed with 0.05% Tween-20/PBS. Samples or recombinant MICA*04, serving as a standard, were added in 7.5% BSA/PBS. After incubation for two hours at 37°C and washing, detection antibody (anti-MICA/B (BAMO3)) was added at a concentration of 1 μg/ml (two hours, 37°C). Plates were washed and incubated with HRP-conjugated anti-mouse IgG2a antibody (1080-05; SouthernBiotech, Birmingham, AL) for one hour at 37°C. After extensive washing, TMB peroxidase substrate (KPL, Gaithersburg, MD) was added and plates were incubated for 35 minutes at room temperature in the dark. HRP activity was stopped by addition of 1 M phosphoric acid and absorbance was measured at 450 nm wavelength.

### Isolation of natural killer cells from healthy donor-derived peripheral blood mononuclear cells

Peripheral blood mononuclear cells were obtained from healthy blood bank donors after informed consent and were isolated using Ficoll density gradient separation. Isolation of natural killer cells was performed using the MACS NK enrichment kit and LS columns, according to the manufacturer's protocol (Miltenyi Biotec, Bergisch Gladbach, Germany) and isolated cells were plated for overnight recovery at 1-2 × 10^6 ^cells/ml in RPMI-1640 supplemented with streptomycin/penicillin and 10% FBS. As determined by flow cytometric analysis, natural killer cell purity always exceeded 88% and T-cell contamination > 0.2% was never detected. For cytokine activation, natural killer cells were cultured in AIM-V medium (Invitrogen), supplemented with 10% pooled human AB-serum (Sanquin, Rotterdam, The Netherlands) and streptomycin/penicillin and stimulated with 10 ng/ml recombinant human interleukin-15 (IL-15) (Bender Medical Systems, Vienna, Austria). Activated natural killer cells were used for Chromium (^51^Cr) release assays within two-four weeks of culture.

### Evaluation of natural killer cell cytotoxicity by Chromium release assays

Cytotoxicity was determined in standard 4-hour ^51^Cr release assays as previously described [20]. For specific natural killer cell receptor (ligand) blocking, natural killer cells or target cells were pre-incubated with blocking antibodies (20 μg/ml; additional file 1, table S1) at room temperature for 20 minutes prior to the ^51^Cr release assay. We previously demonstrated that pre-incubation of natural killer cells with control isotype (IgG1)-matched antibody (anti-CD56) did not affect cytotoxicity [20]. Moreover, pre-incubation of Ewing sarcoma cells with control isotype (IgG1)-matched antibody (anti-CD99; clone B-N24; Diaclone/Sanquin Reagents, Amsterdam, Netherlands) had no effect on natural killer cell-mediated cytolysis (data not shown).

### Pre-treatment of Ewing sarcoma cell lines with histone deacetylase inhibitors

Ewing sarcoma cell lines SK-ES-1, CADO-ES, STA-ET2.1 and TC71 were seeded in 96 well plates at cell densities ranging from 3-15 × 10^3 ^cells/well. Following overnight attachment, cells were incubated for 24 hours with increasing concentrations of specific HDI. Cell viability was measured by 3-(4,5-dimethyl-thiazol-2-yl)-5-(3-carboxymethoxy-phenyl)-2-(4-sulfophenyl)-2H-tetrazolium (MTS) cell viability assay (Promega Benelux, Leiden, The Netherlands). The cytotoxic effect of HDI was quantified by determining IC_50 _values, as defined by the concentration of drug at which 50% of the cells were still metabolically active (table 1). For analysis of phenotypical and functional consequences of HDI treatment, cell lines were pre-treated with defined concentrations of HDI, as indicated in the results section. After 24 hours, cells were harvested, washed and included in flow cytometric or cytotoxicity analyses. In some experiments, cells were pre-incubated for two hours with ATM/ATR inhibitor caffeine (optimal dose based on dose response curve (data not shown)).

**Table 1:** *In vitro *cytotoxicity (IC50 values) of histone deacetylase inhibitors to chemo-sensitive and -resistant EWS cell lines^a^

cell line	NaB (mM)	SAHA (μM)	MS-275 (μM)
chemo-sensitive			

SK-ES-1	1.43	2.85	3.72

TC71	7.85	2.96	30.71

chemo-resistant			

CADO-ES	38.99	3.92	49.97

STA-ET2.1	0.61	1.58	10.06

^a ^'natural' sensitivity of specific cell lines to chemotherapeutic drugs, as previously described [27,35].

### Statistical analyses and artwork

Statistical analyses were performed with SPSS version 16.0 software package. (Paired) t-tests or one-way ANOVA tests were used for comparison of means within or between samples or groups of samples. P < 0.05 was considered statistically significant. Artwork was created using Graphpad Prism 5.0 (La Jolla, CA).

## Results

### Chemotherapy-resistant Ewing sarcoma exhibit reduced susceptibility to lysis by resting natural killer cells

**Figure 1:**
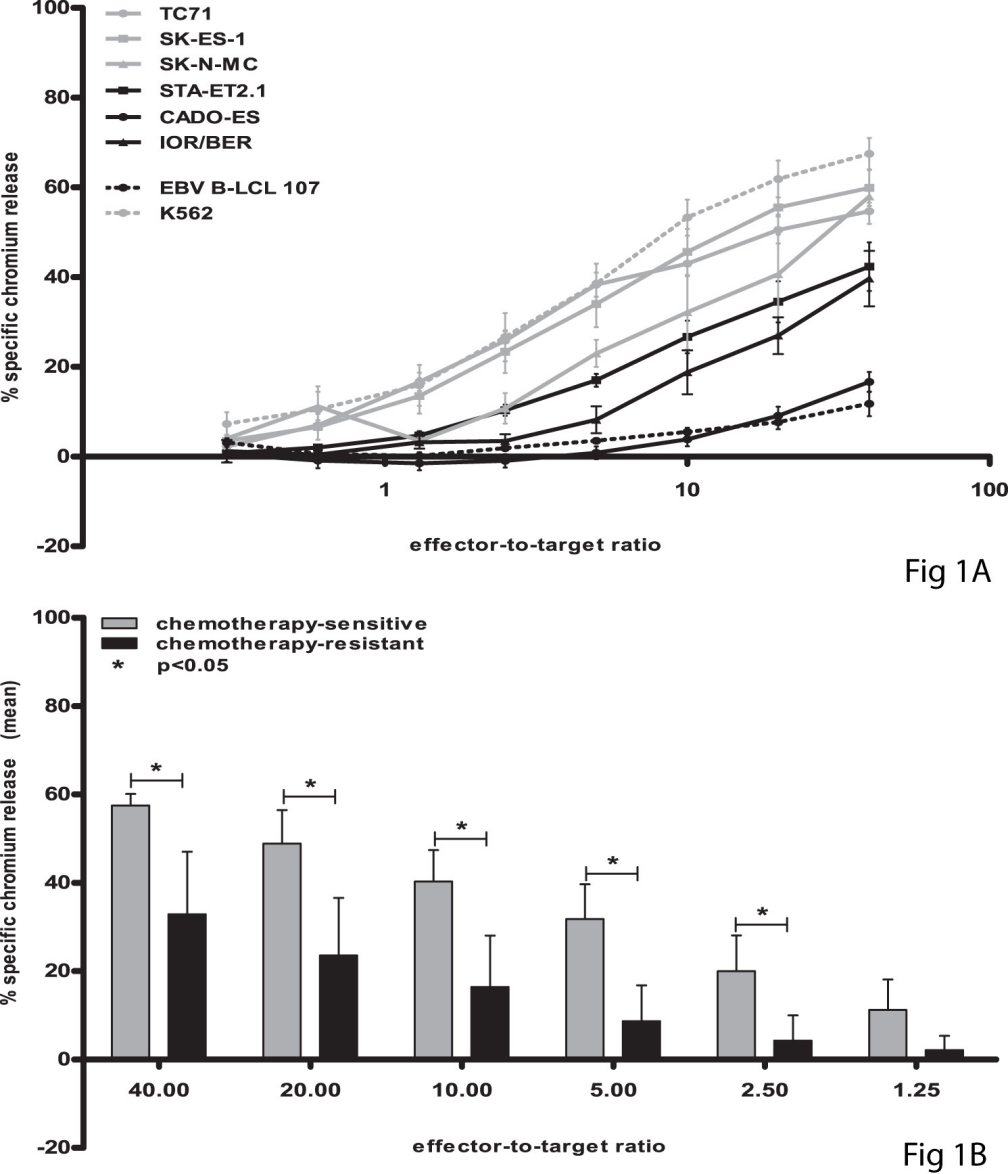
**Chemotherapy-resistant Ewing sarcoma display significantly reduced susceptibility to lysis by resting natural killer cells**. **A-B**. Cytotoxic activity of resting natural killer cells was evaluated in ^51^Cr release assays using chemotherapy-sensitive (grey) and -resistant (black) Ewing sarcoma cell lines TC71 (grey dots), SK-ES-1 (grey squares) and SK-N-MC (grey triangles) respectively STA-ET2.1 (black squares), CADO-ES (black dots) and IOR/BER (black triangles) as target cells. Classical natural killer cell target K562 and EBV B-LCL cell line 107 were used as positive and negative control respectively (A). Results are expressed as the mean ± SEM percentage of specific lysis obtained in at least four independent experiments using different healthy donors. Statistical analysis (t-test) was performed on mean percentages of specific lysis of chemotherapy-sensitive versus -resistant cell lines, revealing significantly reduced sensitivity of chemotherapy-resistant cell lines to resting natural killer cell-mediated cytolysis at all effector-to-target ratio's ≥ 2.5:1 (p < 0.05) (B).

To assess possible cross-resistance of chemotherapy-resistant Ewing sarcoma to natural killer cell cytotoxicity, a panel of both chemotherapy-sensitive (n = 3; TC71, SK-ES-1, SK-N-MC) and -resistant (n = 3; STA-ET2.1, CADO-ES, IOR/BER) cell lines was evaluated for susceptibility to lysis by resting natural killer cells obtained from 4-8 healthy donors. As illustrated in Figure 1A-B, ^51^Cr release assays revealed significantly reduced sensitivity of chemotherapy-resistant cell lines to resting natural killer cell-mediated cytolysis, at effector-to-target ratio's ≥ 2.5:1 (t-test, p < 0.05).

### Comparable expression of ligands for activating and inhibitory natural killer cell receptors in chemotherapy-sensitive and -resistant Ewing sarcoma

**Figure 2:**
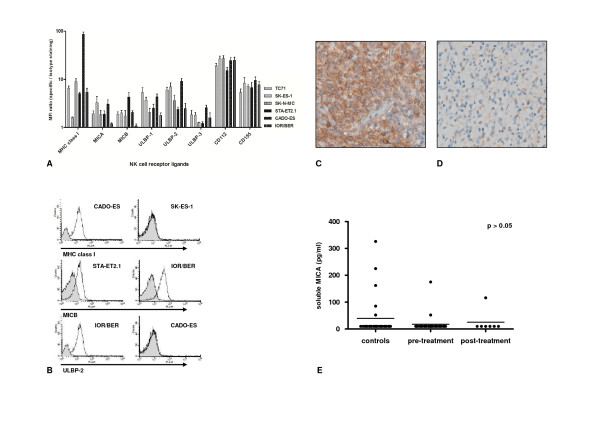
**Non-differential expression of natural killer cell receptor ligands among chemotherapy-sensitive and -resistant Ewing sarcoma *in vitro *or *in vivo***. **A**. Constitutive surface expression of inhibitory (HLA class I) or activating (MICA/B, ULBP1-3, CD112, CD155) natural killer cell receptor ligands in chemotherapy-sensitive (grey; TC71, SK-ES-1 and SK-N-MC) and -resistant (black; CADO-ES, STA-ET2.1 and IOR/BER) Ewing sarcoma cell lines, as assessed by flow cytometry. Results are expressed as the mean ± SD MFI-ratio, obtained in at least two independent experiments. **B**. Representative examples of flow cytometry plots for HLA class I, MICB and ULBP-2 for several Ewing sarcoma cell lines; isotype matched control staining is shown in grey. **C-D**. Light micrographs (20 × magnification) of immunohistochemical stainings for MICA (c; strong expression) and ULBP-1 (d; moderate expression) in sequential primary Ewing sarcoma tumours. **E**. ELISA for detection of soluble MICA in plasma samples obtained from Ewing sarcoma patients (either prior to start of chemotherapy ('pre-treatment') or after completion of therapy ('post-treatment')) and age-comparable controls ('controls'). Statistical analysis (one-way ANOVA) revealed no significant differences in mean soluble MICA levels among 'controls' (39.63 pg/ml), 'pre-treatment' (17.67 pg/ml) or 'post-treatment' (25.14 pg/ml) patients (p > 0.05).

Natural killer cell cytotoxicity to Ewing sarcoma cells critically depends on combined NKG2D and DNAM1 signaling [20,23]. Therefore, constitutive surface expression of ligands for these activating natural killer cell receptors was analyzed in our panel of Ewing sarcoma cell lines by flow cytometry. As represented in Figures 2A-B and additional file 2, figure S1A, flow cytometric analysis revealed no significant differences between chemotherapy-sensitive and -resistant cell lines with regard to expression of ligands for activating natural killer cell receptors NKG2D (MICA, MICB, ULBP1-3) and DNAM1 (CD112, CD155) or killer cell immunoglobulin receptors (HLA class I) (additional file 2, figure S1A, t-test, p > 0.05). Considerable inter-cell line variation, however, was observed for HLA class I expression: chemotherapy-resistant CADO-ES cells, demonstrating substantial resistance to natural killer cells, exhibited 50-fold higher HLA class I expression as compared to chemotherapy-/natural killer cell-sensitive SK-ES-1 cells (Figure 1A and 2A). However, and consistent with previous observations [20], blocking of HLA class I expression could not restore the difference in natural killer cell susceptibility between chemotherapy-sensitive and -resistant cell lines (additional file 2, figure S1B). In addition, and consistent with the observed comparable ligand expression, blocking of NKG2D and DNAM1 on resting natural killer cells in ^51^Cr release assays reduced natural killer cell-mediated cytolysis of both chemotherapy-sensitive and -resistant cell lines to a similar degree (data not shown).

Immunohistochemical staining for expression of NKG2D-ligands MICA and ULBP1 in sequential primary Ewing sarcoma tumour samples obtained from patients with different histological responses to chemotherapy [33] demonstrated abundant expression of (at least one of) these activating ligands throughout all stages of disease and regardless of the histological response to chemotherapy (table 2; Figure 2C-D). In summary, both *in vitro *as well as *in vivo*, natural killer cell receptor ligands are expressed regardless of chemotherapy-sensitivity.

**Table 2:** In vivo NKG2D ligand expression in sequential Ewing sarcoma tumour samples

UPN^a^	sample type (years after diagnosis)	MICA	ULBP1
1	post-chemotherapy resection-GR^b^	n.e.	+

	lung metastasis (2)	++	+

	mediastinal metastasis (3)	++	+

2	pre-treatment biopsy	++	+

	post-chemotherapy resection-GR	+	++

	lung metastasis (5)	+	+/-

	lymph node metastasis (9)	++	n.e.

3	pre-treatment biopsy	+	+

	post-chemotherapy resection-GR	++	+

	lung metastasis (3)	+	++

	lung metastasis (4)	+	n.e.

4	pre-treatment biopsy	+	+

	post-chemotherapy resection-PR^c^	++	+

	bone metastasis (1)	++	-

5	pre-treatment biopsy	++	+/-

	post-chemotherapy resection-PR	++	+

	lung metastasis (1)	++	+/-

6	pre-treatment biopsy	++	-

	post-chemotherapy resection-PR	++	+/-

	lung metastasis (0.5)	++	-

	lung metastasis (2.5)	++	+/-

	lung metastasis (3)	++	+/-

	bone metastasis (3)	++	-

7	post-chemotherapy resection-PR	++	+/-

	lung metastasis (1.5)	++	+

	bone metastasis (2.5)	++	+/-

	lung metastasis (4)	++	+

	lung metastasis (4)	++	+/-

	lung metastasis (5)	++	++

	paravertebral metastasis (5)	++	+

8	pre-treatment biopsy	++	+

	post-chemotherapy resection-GR	+	n.e.

	lung metastasis (8)	++	+

^a^UPN = unique patient number. ^b^GR = good chemotherapy response. ^c^PR = poor chemotherapy

response. n.e. = not evaluable.-= absent, +/- = weak, + = moderate, ++ = strong expression.

### MICA plasma levels are not elevated in Ewing sarcoma patients

Shedding of NKG2D ligands, in particular MICA, from the cell surface represents a mechanism by which tumours escape NKG2D immune surveillance [37-39]. Therefore, and based on the *in situ *expression of MICA in Ewing sarcoma tumours, soluble MICA expression levels were measured in plasma samples from Ewing sarcoma patients. Results were compared to those obtained in plasma samples from age-comparable healthy controls. The majority of plasma samples from healthy controls contained levels of soluble MICA close to the lower detection limit (10 pg/ml) of the assay (median 10 pg/ml; mean 39.63 pg/ml). Plasma concentrations of soluble MICA in patients prior to treatment (median 10 pg/ml; mean 17.67 pg/ml) or after completion of therapy (median 10 pg/ml; mean 25.14 pg/ml) did not significantly differ from those observed in healthy controls (one-way ANOVA, p > 0.05) (Figure 2E).

### Chemotherapy-sensitive and -resistant Ewing sarcoma demonstrate comparable susceptibility to IL-15-activated natural killer cells

**Figure 3:**
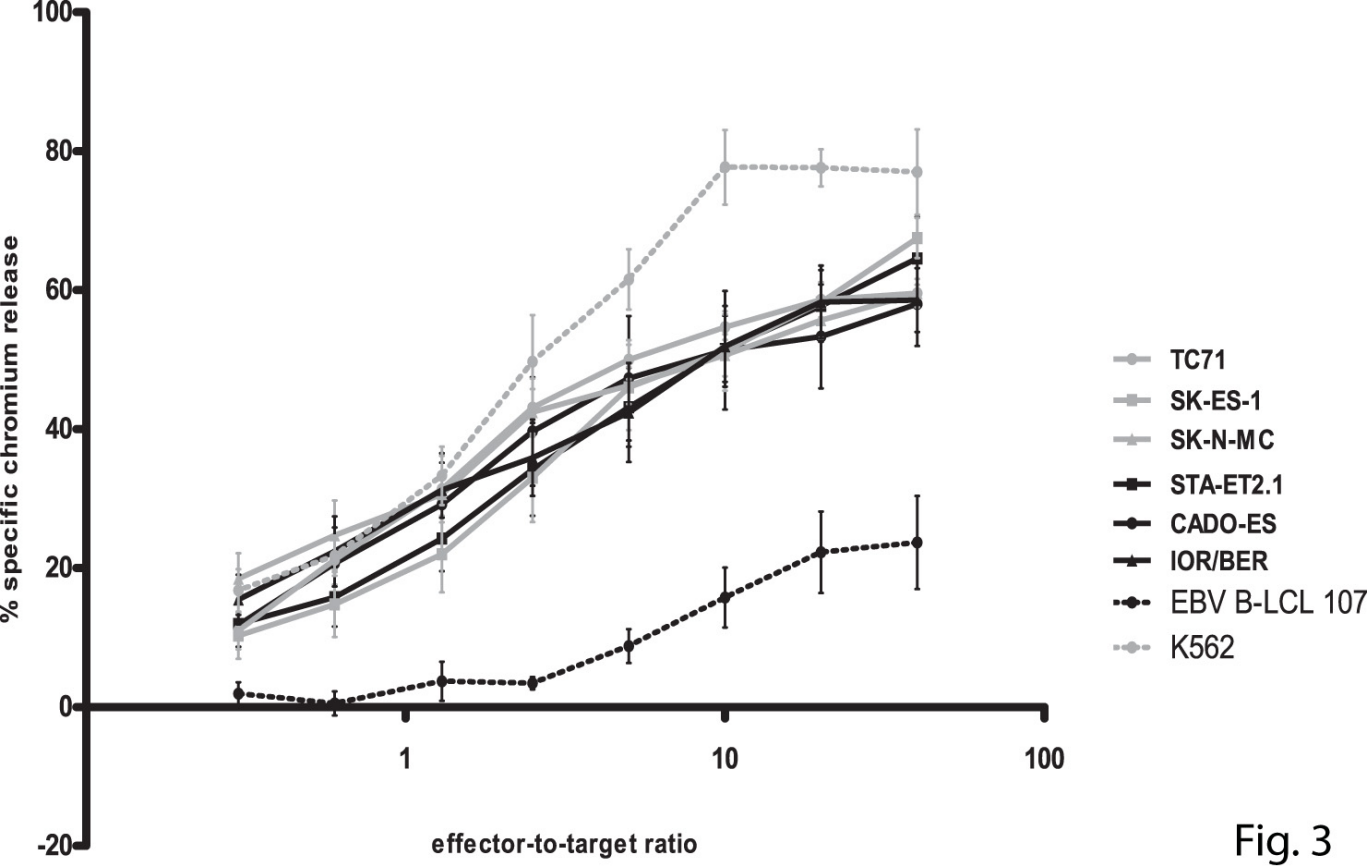
**Activation of natural killer cells with interleukin-15 restores specific cytolysis of chemotherapy-resistant Ewing sarcoma cells to levels similar to those observed for chemotherapy-sensitive cells**. Cytotoxic activity of IL-15-activated natural killer cells was evaluated in ^51^Cr release assays using chemotherapy-sensitive (grey) and -resistant (black) Ewing sarcoma cell lines TC71 (grey dots), SK-ES-1 (grey squares) and SK-N-MC (grey triangles) respectively STA-ET2.1 (black squares), CADO-ES (black dots) and IOR/BER (black triangles) as target cells. K562 and EBV B-LCL cell line 107 were used as positive and negative control respectively. Results are expressed as the mean ± SEM percentages of specific lysis obtained in at least four independent experiments using different healthy donors.

Pre-activation of natural killer cells by either recombinant human IL-15 or co-culture with genetically modified IL-15/4-1BBL expressing K562 feeder cells results in more efficient recognition and lysis of Ewing sarcoma cells [20,23]. Therefore, we evaluated whether the reduced sensitivity of chemotherapy-resistant Ewing sarcoma cell lines to resting natural killer cells could be restored by using IL-15-activated natural killer cells (including cells obtained from donors providing resting natural killer cells). As illustrated in Figure 3, activation of natural killer cells with IL-15 increased specific cytolysis of chemotherapy-resistant Ewing sarcoma to levels similar to those observed for chemotherapy-sensitive cells. Blocking studies using antibodies against NKG2D and DNAM1 revealed comparable contributions of signals provided by these activating natural killer cell receptors to lysis of both chemotherapy-sensitive and -resistant cells (as exemplified for TC71, SK-ES-1, STA-ET2.1 and CADO-ES in additional file 3, figure S2). Together, these data indicate that pre-activation of natural killer cells with IL-15 can overcome resistance of chemotherapy-resistant Ewing sarcoma to natural killer cell-mediated cytolysis.

### Histone deacetylase inhibitors induce NKG2D ligand expression in Ewing sarcoma in an ATM/ATR-dependent manner

To assess the capacity of HDI to sensitize Ewing sarcoma for immune-mediated cytotoxicity, cell lines were exposed for 24 hours to HDI belonging to three different structural classes: hydroxamic acid SAHA, short-chain fatty acid NaB and benzamide MS-275. Analysis of the direct cytotoxic effects of these drugs by cell viability assays revealed variation in the sensitivity of the different cell lines to these agents, as represented by variances in drug-specific IC_50 _values (table 1).

**Figure 4:**
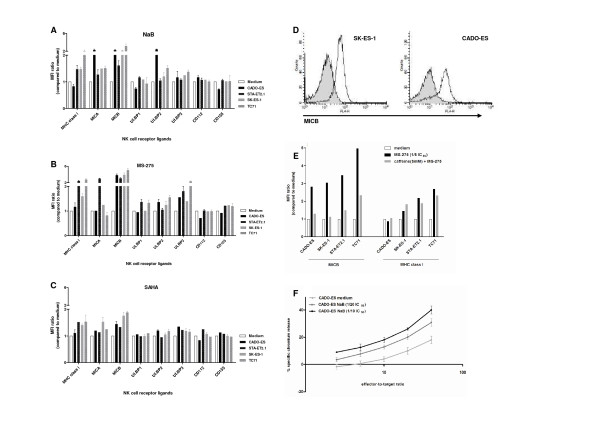
**Histone deacetylase inhibitor-induced, ATM/ATR-dependent NKG2D ligand expression sensitizes Ewing sarcoma for natural killer cell cytotoxicity**. **A-C**. Heterogeneous induction of activating NKG2D ligands and/or inhibitory HLA class I molecules in chemotherapy-sensitive (grey) and -resistant (black) cell lines upon pre-treatment with HDI NaB (A), MS-275 (B) and SAHA (C), as assessed by flow cytometry. Results are expressed as mean ± SEM fold increase in MFI-ratio over medium control, obtained in at least three independent experiments. [HDI] = 1/5 IC_50 _value, except for CADO-ES (NaB 1/20 IC_50 _value; MS-275 and SAHA 1/10 IC_50 _value). **D**. Representative examples of flow cytometry plots for MICB for SK-ES-1 and CADO-ES upon pre-treatment with HDI MS-275 (IC_50 _values); untreated cells in grey. **E**. Pre-treatment with caffeine (5 mM) for 2 hours prior to incubation with HDI MS-275 (1/5 of IC_50 _value) largely abolished HDI-mediated MICB expression (similar results were observed for MICA and ULBP2-3), as assessed by flow cytometry. No such effects were observed for HLA class I expression. Results are expressed as the mean ± SEM fold increase in MFI-ratio over medium control, and are representative of at least two independent experiments. Similar results were obtained for NaB and SAHA (not shown). **F**. Cytotoxicity of resting natural killer cells was evaluated in ^51^Cr release assays. Pre-treatment of CADO-ES cells with NaB (1/20 and 1/10 IC_50 _value) sensitized, in a dose-dependent manner, for natural killer cell cytotoxicity. Statistical analysis (paired t-test) revealed significantly increased sensitivity of NaB pre-treated cells at effector-to-target ratio's > 5:1 (p < 0.05). Similar results were observed for CADO-ES with MS-275 and SAHA, and for SK-ES-1 with SAHA (not shown).

Flow cytometric evaluation of natural killer cell receptor ligand expression upon 24-hour pre-treatment of the cell lines with defined (IC_50_-related) concentrations of HDI revealed heterogeneous but consistent induction of several activating NKG2D ligands, in particular MICB, in all cell lines evaluated. The most pronounced effects were observed upon pre-treatment with NaB and MS-275, resulting in up to five-fold induction of MICB (Figure 4A-D). Expression of activating DNAM1 ligands (CD112 and CD155) remained largely unchanged. Induction of HLA class I expression was detectable in cell lines STA-ET2.1 and TC71, whereas in CADO-ES, with relatively high levels of constitutive HLA class I expression (Figure 2A), no induction was observed. Induction of HLA class I expression was demonstrated in the SK-ES-1 cell line as well. However, since constitutive HLA class I expression was hardly detectable in this cell line (Figure 2A), the observed less than two-fold induction by histone deacetylase inhibitors still resulted in marginal HLA class I expression (Figure 4A-C).

In addition to a direct epigenetic effect resulting in increased expression of target genes, HDI may induce activation of the ATM/ATR-mediated DNA damage response which, in turn, induces NKG2D ligand expression [16]. To assess whether the ATM/ATR response pathway contributed to HDI-induced expression of NKG2D ligands in Ewing sarcoma cells, cell lines were treated with ATM/ATR inhibitor caffeine (5 mM) for 2 hours prior to incubation with these agents. Indeed, caffeine pre-treatment largely prevented HDI-mediated induction of NKG2D ligands, but not HLA class I, in all cell lines (as exemplified for MICB/HLA class I expression upon MS-275 in Figure 4E).

### Histone deacetylase inhibitors sensitize Ewing sarcoma for NKG2D-dependent natural killer cell cytotoxicity

To assess the functional relevance of HDI-induced alterations in natural killer cell receptor ligand expression, HDI pre-treated Ewing sarcoma cells were subjected to ^51^Cr release assays using resting natural killer cells. Pre-treatment of the chemotherapy-resistant CADO-ES cell line with HDI NaB, MS-275 and SAHA sensitized, in a dose-dependent manner, for natural killer cell cytotoxicity (as shown for NaB in Figure 4F; paired t-test (medium versus 1/20 of IC_50 _value), p < 0.05 at effector-to-target ratio's ≥ 5:1). Similarly, pre-treatment of SK-ES-1 cells with SAHA (1/5 of IC_50 _value), but not NaB or MS-275, resulted in significantly increased cytolysis (data not shown). In cell lines TC71 and STA-ET2.1, despite induction of activating NKG2D ligands, no sensitization for natural killer cell cytotoxicity was detectable (additional file 4, figure S3A). The role of NKG2D in natural killer cell recognition and cytolysis of HDI-treated Ewing sarcoma cells was evaluated by performing ^51^Cr release assays in the presence of a blocking antibody against NKG2D. In both CADO-ES and SK-ES-1 cells, these experiments revealed (persistent) dependence on signaling via this activating natural killer cell receptor since blocking reduced lysis of both untreated and HDI-treated cells (as demonstrated for CADO-ES upon NaB in additional file 4, figure S3B).

## Discussion

Despite current multimodal therapy consisting of high-dose chemotherapy, surgery and radiotherapy, survival of patients with Ewing sarcoma has not improved during the past decade. Integration of natural killer cell-based combination immunotherapy into first-line treatment regimens or as a second-line approach, as recently emphasized by Ahn et al. [26], represents a promising treatment option for Ewing sarcoma, in particular for patients with either intrinsic or acquired therapy-resistance. Current immunotherapeutic strategies presume therapy-resistant tumours to be sensitive to immune-mediated cytotoxicity. However, depending on the pathways involved, resistance to conventional therapies might render cells cross-resistant to immunotherapy [28]. The observed reduced susceptibility of chemotherapy-resistant Ewing sarcoma to resting natural killer cell cytotoxicity might be indicative for the existence of cross-resistance mechanisms.

Resistance of Ewing sarcoma to chemotherapy correlates (inversely) with differential expression of genes involved in apoptosis, including caspase-8 and p53 pathways [27,40,41]. Based on available data about caspase-8 expression and p53 mutation status in our panel of cell lines [27,34,36,42-44], however, the observed cross-resistance to resting natural killer cells cannot be explained by aberrant expression or the mutation status of these proteins alone. P-glycoprotein is a drug-efflux pump that confers multidrug resistance to a wide range of chemotherapeutic drugs, including key chemotherapeutic agents used for Ewing sarcoma treatment [4,45]. In addition to its ability to efflux drugs, P-glycoprotein regulates apoptosis by inhibition of caspase activation [46]. Theoretically, P-glycoprotein expression in our panel of cell lines might contribute to the observed (relative) cross-resistance to natural killer cells [47,48]. However, whereas caspase-dependent apoptosis is prevented, P-glycoprotein expressing cells remain sensitive to perforin/granzyme B-induced cell death [46]. We previously demonstrated natural killer cell-mediated apoptosis induction in Ewing sarcoma cells to be largely dependent on the perforin/granzyme B-mediated granule exocytosis pathway rather than on caspase-dependent death receptor pathways [36]. Therefore, we assume P-glycoprotein expression a non-crucial factor in cross-resistance of Ewing sarcoma to immune-mediated cytotoxicity. Since susceptibility of Ewing sarcoma to natural killer cell-mediated lysis depends on expression of ligands for activating and inhibitory natural killer cell receptors [20,23,24], differential expression of these ligands among chemotherapy-sensitive and -resistant cells could be an alternative explanation for the observed chemo-/immunotherapy cross-resistance of Ewing sarcoma. Considerable inter-cell line variation was observed for HLA class I expression, with a 50-fold increased expression in chemotherapy- and natural killer cell-resistant CADO-ES cells as compared to chemotherapy-/natural killer cell-sensitive SK-ES-1 cells. In a recent report, Holmes et al. [24] point to the existence of natural killer cell activation/inhibition thresholds that allow small changes in HLA class I cell surface expression to dramatically alter susceptibility of Ewing sarcoma to natural killer cells. However, no significant effects of HLA class I blocking were observed (current and previous studies [20]), excluding a major contribution of dominant inhibitory signals provided by (differential) HLA class I expression. Moreover, and in addition to the observed comparable constitutive expression of activating NKG2D and DNAM1-ligands, blocking of NKG2D and DNAM1 on resting natural killer cells reduced natural killer cell-mediated cytolysis of both chemotherapy-sensitive and -resistant cells to a similar degree. Together, our results point to the existence of true cross-resistance to immune-mediated cytolysis rather than to dissimilar expression of activating/inhibitory natural killer cell receptor ligands or differential dependency on signaling via activating receptors NKG2D and DNAM1. As yet, since ligands for activating NCR are largely unknown [7,24,49], a possible contribution of such ligand can not be excluded. Similary, other possible causes for cross-resistance to conventional and immunotherapeutic approaches in Ewing sarcoma, that have not been addressed (e.g. (over)expression of (other) pro- or anti-apoptotic factors [50] or dysregulation of specific signaling pathways [51]), may need further investigation.

Importantly, the relative resistance of chemotherapy-resistant Ewing sarcoma cells to lysis by resting natural killer cells could be overcome by pre-activation of natural killer cells with IL-15. Although the exact mechanisms underlying cross-resistance to chemotherapeutic drugs and immune-mediated cytotoxicity have not been fully elucidated, utilization of complementary (apoptotic) pathways or stronger pro-apoptotic signals by cytokine-activated natural killer cells [52] may contribute to the observed increased cytotoxic potential of these cells toward (chemotherapy-resistant) Ewing sarcoma. Irrespective of the exact mechanism, however, the observation that chemotherapy-resistant Ewing sarcoma do not exhibit cross-resistance to IL-15-activated natural killer cell-mediated immunotherapy suggests that cytokine-activated natural killer cell therapies may represent promising immunotherapeutic options for patients with Ewing sarcoma.

Due to the nature of this (bone) tumour, attempts to establish primary tumour cell cultures from therapy-naive biopsies for evaluation of natural killer cell cytotoxicity towards these targets have so far been unsuccessful. Support for potential *in vivo *immune recognition of Ewing sarcoma tumours, however, was provided by immunohistochemical analysis of sequential Ewing sarcoma tumour samples demonstrating expression of (at least one of the) activating natural killer cell receptor ligands MICA and ULBP1 throughout all stages of disease and regardless of the histological response to chemotherapy. Moreover, and similar to previous observations in leukemia [10], a potential role for natural killer cell alloreactivity in Ewing sarcoma disease control has been suggested [25]. In addition to recognition by natural killer cells, expression of NKG2D ligands might improve anti-tumour immune responses by specific T-lymphocyte subsets [53]. We recently reported on the (prognostically beneficial) impact of tumour-infiltrating T-lymphocytes on tumour progression in therapy-naive Ewing sarcoma [54]. Despite *in situ *expression of NKG2D ligands, tumour cells may escape from NKG2D immunosurveillance by (enhanced) shedding of these ligands from their cell surfaces. Subsequently, circulating tumour-derived soluble ligands may cause downregulation of NKG2D and, in turn, severely impair cytotoxic effector functions of both T- and natural killer cells [37-39]. Based on our current results demonstrating non-elevated soluble MICA levels in plasma of Ewing sarcoma patients (as compared to age-comparable healthy controls) as well as our previous observation of intact NKG2D expression on natural killer cells of these patients [20], we do not consider NKG2D ligand shedding a relevant immune escape mechanism in patients with this tumour. We previously reported on reduced levels of HLA class I expression in advanced-stage Ewing sarcoma as a potential immune escape mechanism hampering recognition by tumour-reactive T-lymphocytes and feasibility of T cell-based immunotherapeutic approaches [55]. Our current observations of 1) *in situ *expression of ligands for activating natural killer cell receptors throughout all stages of disease, including (chemotherapy-resistant) metastatic disease, 2) the absence of (enhanced) shedding of these activating natural killer cell receptor ligands, 3) the above mentioned reduced expression of inhibitory HLA class I molecules in advanced-stage Ewing sarcoma cases and 4) the observed susceptibility of (chemotherapy-resistant) Ewing sarcoma to IL-15-activated natural killer cells further support the potential of Ewing sarcoma as an attractive target for natural killer cell-based immunotherapy.

Several phase I-III clinical trials now prove HDI-treatment to be safe and effective in both hematological and solid tumours (as reviewed by [56]) and recently, different HDI were approved by the US Food and Drug Administration for use in patients [57]. With regard to Ewing sarcoma, a role for HDI in reversal of oncogenic transcriptional repression has been proposed [58]. In addition, HDI-mediated cytotoxicity has been demonstrated both *in vitro *and *in vivo *and sensitization for both conventional and more experimental treatment modalities has been suggested [29,31,32]. Here, we demonstrate the ability of three structurally different HDI to, at doses 5-20 times lower than the established IC50 values, improve potential immune recognition (by natural killer cells and/or specific T-lymphocyte subsets) of both chemotherapy-sensitive and -resistant Ewing sarcoma. HDI-pretreatment resulted in ATM/ATR-dependent induction of NKG2D ligands, in particular MICA and MICB, in all cell lines. Moreover, dose-dependent sensitization for natural killer cell cytotoxicity was observed in 2/4 cell lines, including the chemotherapy-resistant CADO-ES cell line. The doses used *in vitro *were below or within the range of the maximum tolerated dose as determined in recent clinical trials (in children) [59,60]. Natural killer cell cytotoxicity depended on NKG2D-NKG2D ligand interactions, since blocking of NKG2D abrogated cytolysis. No sensitization was observed for TC71 and STA-ET2.1 cells, despite induction of NKG2D ligands. As yet, an adequate explanation for this observation is lacking. Although HDI-pretreatment induced HLA class I in these cell lines, a contribution of dominant inhibitory signals provided by this increased expression seems unlikely since HLA class I blocking did not significantly affect natural killer cell-mediated cytolysis.

Collectively, our data provide a rationale for combination immunotherapy involving immune effector cell (IL-15-activated natural killer cells) and target cell (HDI) manipulation in first- and/or second-line treatment regimens for Ewing sarcoma.

## Conclusions

Patients with Ewing sarcoma (EWS) have a poor prognosis, despite current multimodal therapy. Integration of immunotherapeutic strategies, including natural killer (NK) cell-based therapies, into first-line treatment regimens or introduction of these approaches as second-line therapy may represent promising treatment options. *Ex vivo *immune cell activation and/or (simultaneous) sensitization of target cells for immune-mediated killing by combination immunotherapy may overcome intrinsic/acquired resistance to conventional therapies and improve (immuno)therapeutic efficacy. Here, we provide the first evidence that combination immunotherapy using histone deacetylase inhibitors and (interleukin-15-activated) NK cells improves immune recognition of both therapy-sensitive and -resistant EWS and sensitizes for NK cell cytotoxicity. *In vivo *expression of activating NK cell receptor ligands throughout all disease-stages, regardless of chemotherapy-response, supports their potential *in vivo *role in immune recognition of EWS. Our data provide a rationale for combination immunotherapy involving simultaneous immune cell (interleukin-15-activated NK cells) and target cell (histone deacetylase inhibitors) manipulation in first-/second-line treatment regimens for EWS.

## Competing interests

The authors declare that they have no competing interests.

## Authors' contributions

All authors contributed to conception and/or design of the study. DB, HIV, SJS and SK conducted experiments and performed data analyses. DB, MWS, EPB, PCWH and ACL were involved in interpretation of data. All authors were involved in drafting and/or critical revision of the manuscript and approved the final submitted version.

## Supplementary Material

Additional file 1 **Antibodies used for flow cytometry, NK cell receptor (ligand) blocking, immunohistochemistry and ELISA**. Click here for file

Additional file 2 **A. Constitutive surface expression of inhibitory (HLA class I) or activating (MICA/B, ULBP1-3, CD112, CD155) natural killer cell receptor ligands in chemotherapy-sensitive (grey) and -resistant (black) Ewing sarcoma cell lines, as assessed by flow cytometry**. Results are expressed as the mean ± SD MFI-ratio, obtained in at least two independent experiments. Statistical analysis (t-test) was performed on mean MFI-ratio's (for each ligand) of chemotherapy-sensitive versus -resistant cell lines, revealing no significant differences in expression levels of these ligands (p > 0.05). **B**. Cytotoxic activity of resting natural killer cells was evaluated in ^51^Cr release assays using chemotherapy-sensitive (TC71 (blue), SK-ES-1 (purple), SK-N-MC (pink)) and -resistant (STA-ET-2.1 (black), CADO-ES (dark grey), IOR/BER (light grey)) Ewing sarcoma cell lines as target cells. Ewing sarcoma cells were either left untreated (solid bars) or pre-incubated with HLA class I blocking antibody DX17 (checked bars). Results are expressed as the mean ± SD percentage of specific lysis obtained in at least two independent experiments using different healthy donors. Click here for file

Additional file 3 **Cytotoxic activity of IL-15-activated natural killer cells was evaluated in ^51^Cr release assays using chemotherapy-sensitive (TC71 (blue), SK-ES-1 (purple)) and -resistant (STA-ET-2.1 (black), CADO-ES (grey) Ewing sarcoma cell lines as target cells**. Ewing sarcoma cells were either left untreated (solid bars) or pre-incubated with NKG2D and DNAM-1 blocking antibodies (checked bars). Results are expressed as the mean ± SD percentage of specific lysis obtained in at least two independent experiments using different healthy donors. Click here for file

Additional file 4 **A. Cytotoxicity of resting natural killer cells was evaluated in ^51^Cr release assays using MS-275-pretreated TC71 and STA-ET2.1 cells**. Despite induction of activating NKG2D ligands, no sensitization for natural killer cell cytotoxicity was detectable (at doses up to 1/5 of IC_50 _value). Similar results were observed for both cell lines upon pre-treatment with NaB and SAHA (not shown). Results are expressed as the mean ± SEM percentages of specific lysis obtained in at least two independent experiments using different healthy donors. **B**. Upon HDI-pretreatment, persistent dependency of resting natural killer cell-mediated cytotoxicity on signaling via activating receptor NKG2D was demonstrated when ^51^Cr release assays were performed in the presence of a blocking antibody against NKG2D. Blocking reduced resting natural killer cell-mediated lysis of both untreated and HDI pre-treated cells to similar levels, as demonstrated for CADO-ES upon pre-treatment with NaB. Similar results were obtained for CADO-ES with MS-275 and SAHA, as well as for SK-ES-1 with SAHA (not shown). K562 and EBV B-LCL cell line 107 were used as positive and negative control respectively (not shown). Results are expressed as the mean ± SEM percentages of specific lysis obtained in at least two independent experiments using different healthy donors. Click here for file
