# Supplementary material for: Histone deacetylase inhibitors enhance expression of NKG2D ligands in Ewing sarcoma and sensitize for natural killer cell-mediated cytolysis
Source: Clin Sarcoma Res. 2012 Feb 8;2:8. doi: 10.1186/2045-3329-2-8 (PMC3351702; doi:10.1186/2045-3329-2-8)
Supplement: Additional file 1 — Antibodies used for flow cytometry, NK cell receptor (ligand) blocking, immunohistochemistry and ELISA. [file 2045-3329-2-8-S1.DOC]

| Table S1. Antibodies (Ab) used for flow cytometry, NK cell receptor (ligand) blocking, immunohistochemistry and ELISA | | | |
| --- | --- | --- | --- |
| application | Ab | clone | source |
|  |  |  |  |
| flow cytometry |  |  |  |
|  |  |  |  |
|  | anti-HLA-A/B/C-FITC | G46-2.6 | BD Pharmingen, San Diego, CA |
|  | anti-MICA | 159227 | R&D Systems, Abingdon, United Kingdom |
|  | anti-MICB | 236511 | R&D Systems, Abingdon, United Kingdom |
|  | anti-ULBP1 | 170818 | R&D Systems, Abingdon, United Kingdom |
|  | anti-ULBP2 | 165903 | R&D Systems, Abingdon, United Kingdom |
|  | anti-ULBP3 | 166510 | R&D Systems, Abingdon, United Kingdom |
|  | anti-CD112-PE | R2.477.1 | Beckman Coulter Immunotech, Marseille, France |
|  | anti-CD155 | PV.404 | Beckman Coulter Immunotech, Marseille, France |
|  | anti-CD3-PerCPCy5.5 | SK7 | BD Pharmingen, San Diego, CA |
|  | anti-CD56-PE | N901 | Beckman Coulter Immunotech, Marseille, France |
|  | anti-CD20-FITC | L27 | BD Pharmingen, San Diego, CA |
|  | anti-CD14-APC | MФP9 | BD Pharmingen, San Diego, CA |
|  | APC goat anti-mouse Ig |  | BD Pharmingen, San Diego, CA |
|  |  |  |  |
| NK cell receptor (ligand) blocking |  |  |  |
|  | anti-HLA-A/B/C | DX17 | BD Pharmingen, San Diego, CA |
|  | anti-NKG2D | 149810 | R&D Systems, Abingdon, United Kingdom |
|  | anti-DNAM1 | DX11 | BD Pharmingen, San Diego, CA |
|  |  |  |  |
| immunohistochemistry |  |  |  |
|  | anti-MICA | AF1300 | R&D Systems, Abingdon, United Kingdom |
|  | anti-ULBP1 | HPA007547 | Sigma-Aldrich, Zwijndrecht, The Netherlands |
|  |  |  |  |
| ELISA |  |  |  |
|  | anti-MICA | AMO1 | Axxora Deutschland GmbH, Lörrach, Germany |
|  | anti-MICA/B | BAMO3 | Axxora Deutschland GmbH, Lörrach, Germany |
|  | anti-mouse IgG2a-HRP | 1080-05 | SouthernBiotech, Birmingham, AL |
|  |  |  |  |
