# Supplementary figures and images for: Histone deacetylase inhibitors enhance expression of NKG2D ligands in Ewing sarcoma and sensitize for natural killer cell-mediated cytolysis
Source: Clin Sarcoma Res. 2012 Feb 8;2:8. doi: 10.1186/2045-3329-2-8 (PMC3351702; doi:10.1186/2045-3329-2-8)

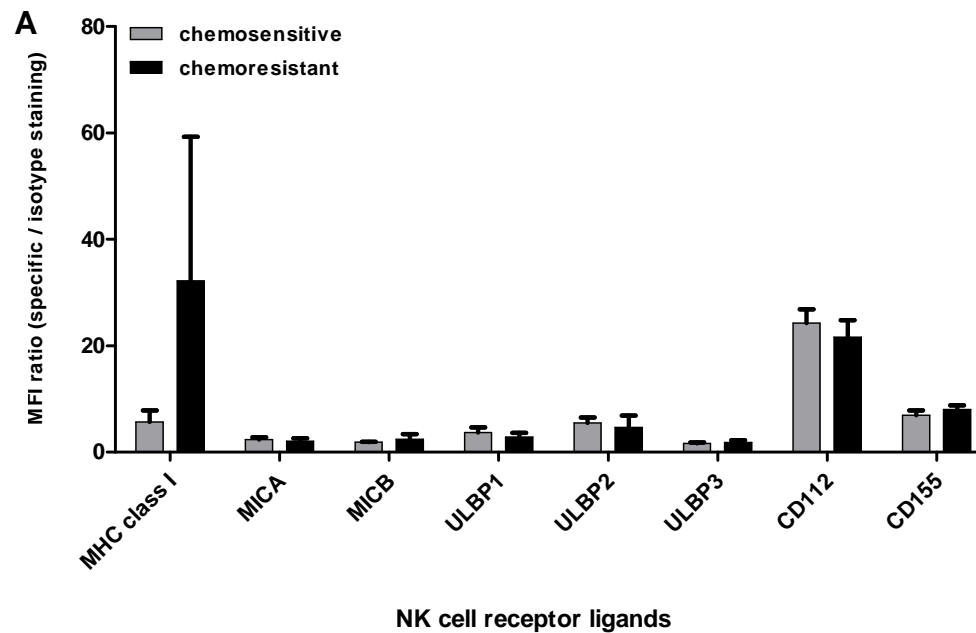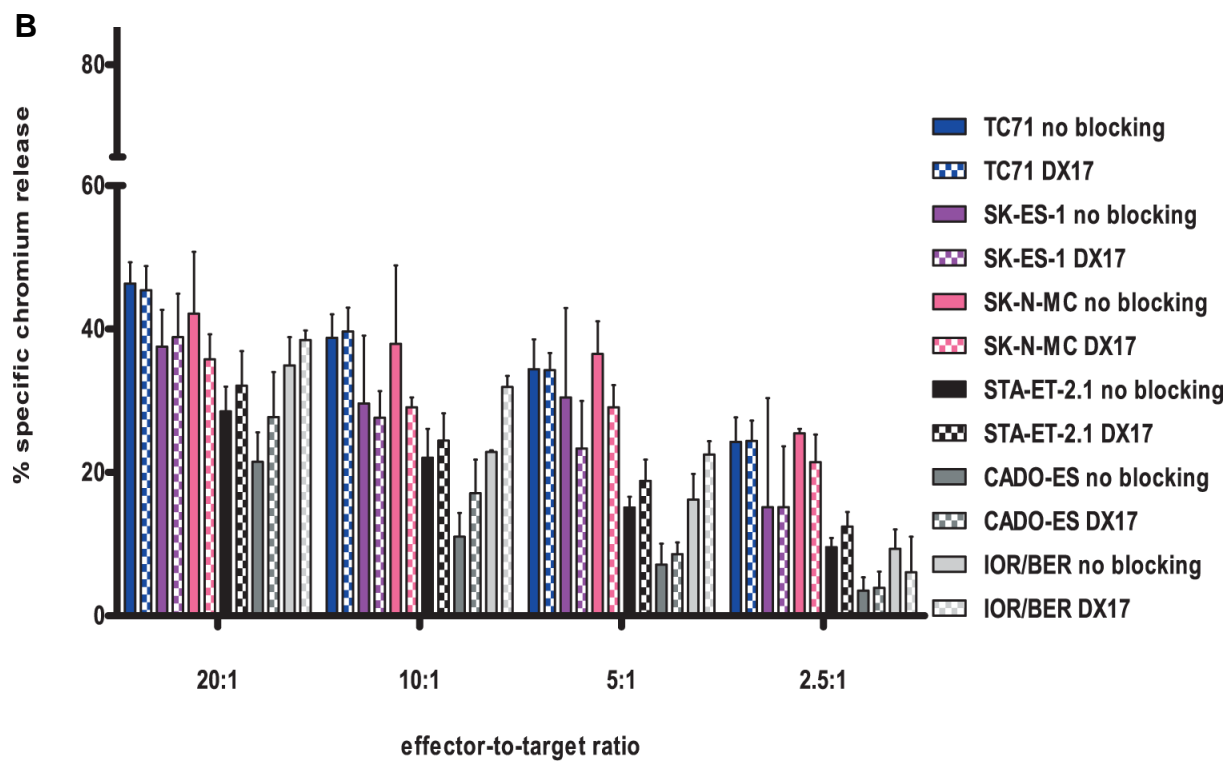

Supplement: Additional file 2 — A. Constitutive surface expression of inhibitory (HLA class I) or activating (MICA/B, ULBP1-3, CD112, CD155) natural killer cell receptor ligands in chemotherapy-sensitive (grey) and -resistant (black) Ewing sarcoma cell lines, as assessed by flow cytometry. Results are expressed as the mean ± SD MFI-ratio, obtained in at least two independent experiments. Statistical analysis (t-test) was performed on mean MFI-ratio's (for each ligand) of chemotherapy-sensitive versus -resistant cell lines, revealing no significant differences in expression levels of these ligands (p > 0.05). B. Cytotoxic activity of resting natural killer cells was evaluated in 51Cr release assays using chemotherapy-sensitive (TC71 (blue), SK-ES-1 (purple), SK-N-MC (pink)) and -resistant (STA-ET-2.1 (black), CADO-ES (dark grey), IOR/BER (light grey)) Ewing sarcoma cell lines as target cells. Ewing sarcoma cells were either left untreated (solid bars) or pre-incubated with HLA class I blocking antibody DX17 (checked bars). Results are expressed as the mean ± SD percentage of specific lysis obtained in at least two independent experiments using different healthy donors. [file 2045-3329-2-8-S2.PDF]

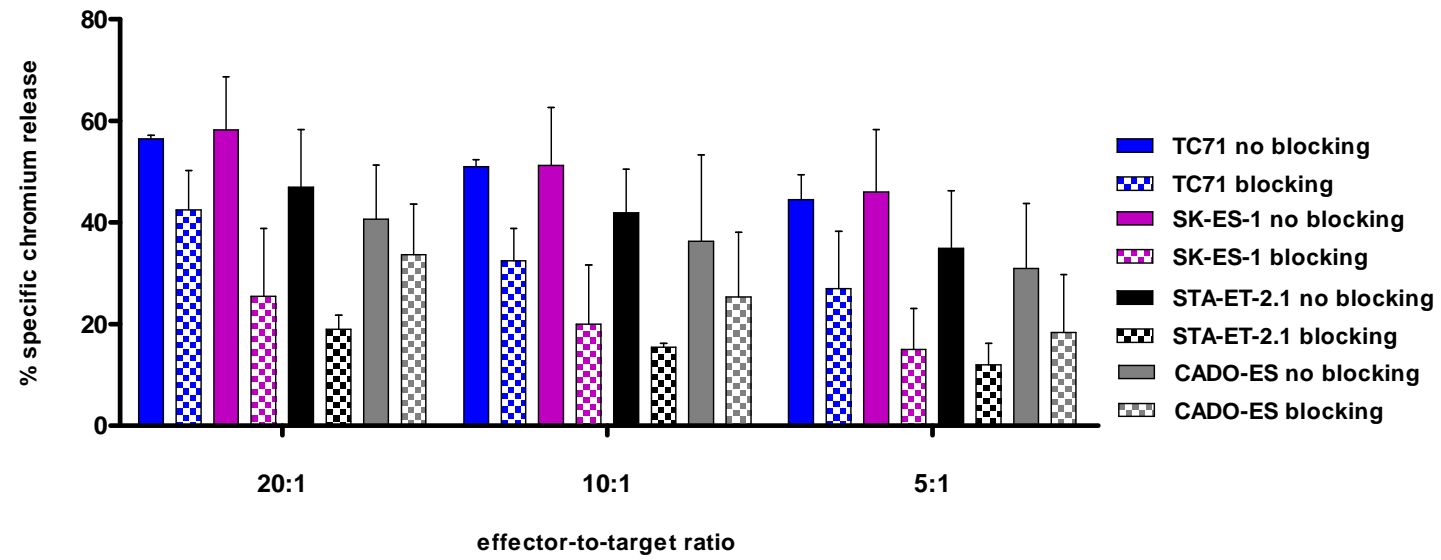

Supplement: Additional file 3 — Cytotoxic activity of IL-15-activated natural killer cells was evaluated in 51Cr release assays using chemotherapy-sensitive (TC71 (blue), SK-ES-1 (purple)) and -resistant (STA-ET-2.1 (black), CADO-ES (grey) Ewing sarcoma cell lines as target cells. Ewing sarcoma cells were either left untreated (solid bars) or pre-incubated with NKG2D and DNAM-1 blocking antibodies (checked bars). Results are expressed as the mean ± SD percentage of specific lysis obtained in at least two independent experiments using different healthy donors. [file 2045-3329-2-8-S3.PDF]

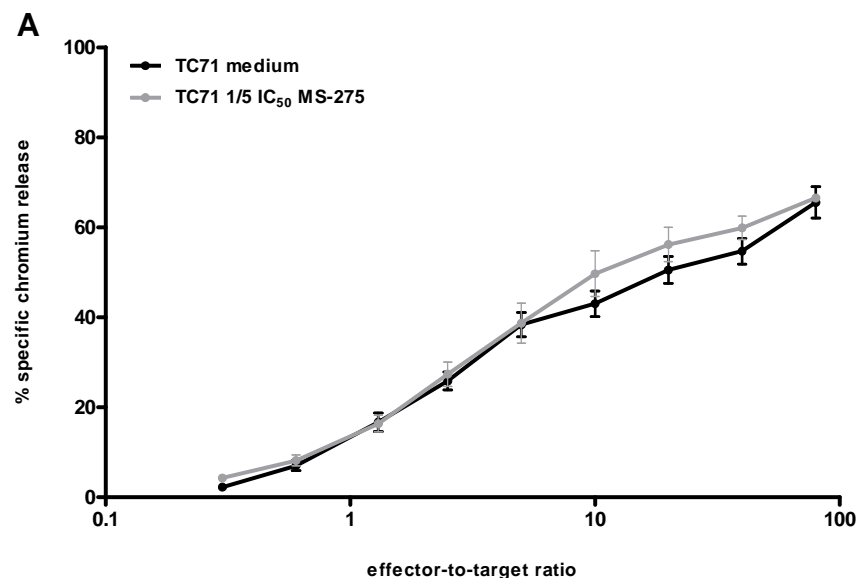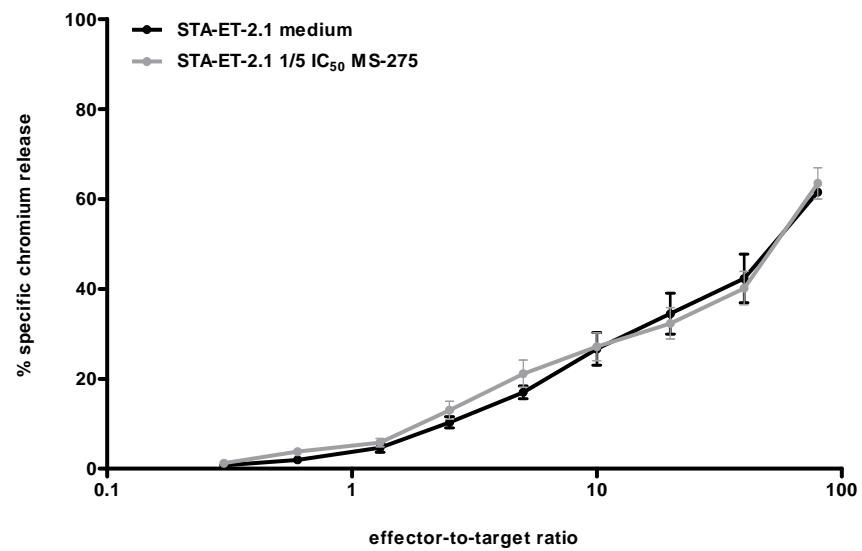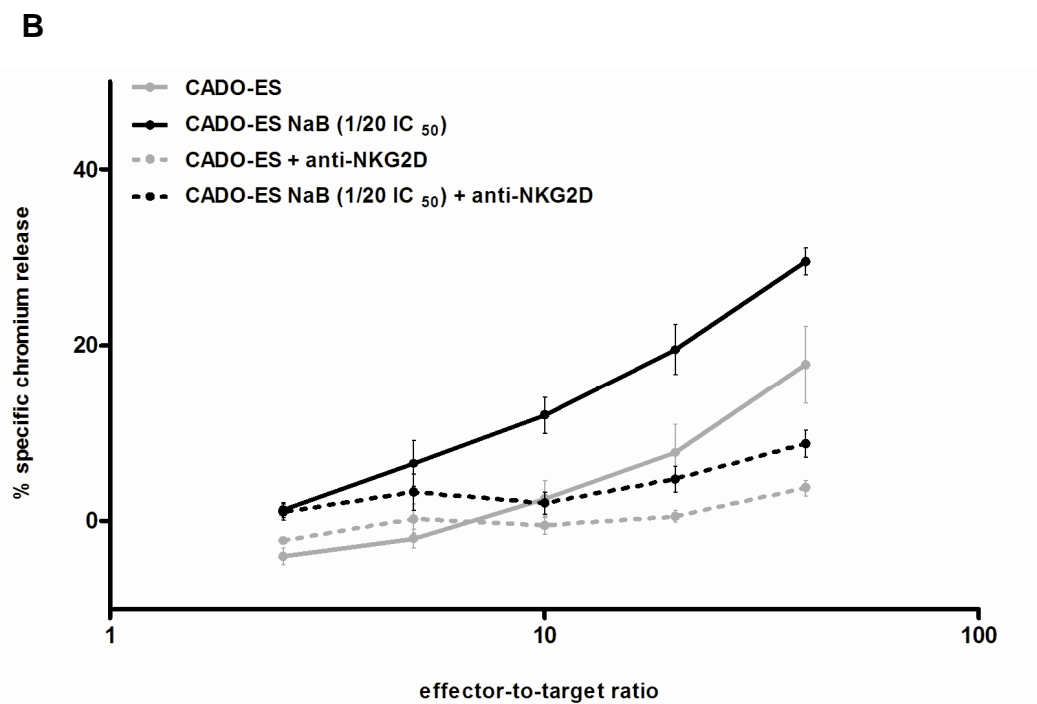

Supplement: Additional file 4 — A. Cytotoxicity of resting natural killer cells was evaluated in 51Cr release assays using MS-275-pretreated TC71 and STA-ET2.1 cells. Despite induction of activating NKG2D ligands, no sensitization for natural killer cell cytotoxicity was detectable (at doses up to 1/5 of IC50 value). Similar results were observed for both cell lines upon pre-treatment with NaB and SAHA (not shown). Results are expressed as the mean ± SEM percentages of specific lysis obtained in at least two independent experiments using different healthy donors. B. Upon HDI-pretreatment, persistent dependency of resting natural killer cell-mediated cytotoxicity on signaling via activating receptor NKG2D was demonstrated when 51Cr release assays were performed in the presence of a blocking antibody against NKG2D. Blocking reduced resting natural killer cell-mediated lysis of both untreated and HDI pre-treated cells to similar levels, as demonstrated for CADO-ES upon pre-treatment with NaB. Similar results were obtained for CADO-ES with MS-275 and SAHA, as well as for SK-ES-1 with SAHA (not shown). K562 and EBV B-LCL cell line 107 were used as positive and negative control respectively (not shown). Results are expressed as the mean ± SEM percentages of specific lysis obtained in at least two independent experiments using different healthy donors. [file 2045-3329-2-8-S4.PDF]
